# Analysis of the Exposure of Organisms to the Action of Nanomaterials

**DOI:** 10.3390/ma13020349

**Published:** 2020-01-12

**Authors:** Anita Staroń, Olga Długosz, Jolanta Pulit-Prociak, Marcin Banach

**Affiliations:** Faculty of Chemical Engineering and Technology, Cracow University of Technology, 24 Warszawska St., 31-155 Cracow, Poland; anilos@chemia.pk.edu.pl (A.S.); OlgaDlugosz@interia.pl (O.D.); jolantapulit@indy.chemia.pk.edu.pl (J.P.-P.)

**Keywords:** metal nanoparticles, metal oxide nanoparticles, medicine, reducing toxicity, exposure, risk assessment

## Abstract

The rapid development of the production of materials containing metal nanoparticles and metal oxides is a potential risk to the environment. The degree of exposure of organisms to nanoparticles increases from year to year, and its effects are not fully known. This is due to the fact that the range of nanoparticle interactions on cells, tissues and the environment requires careful analysis. It is necessary to develop methods for testing the properties of nanomaterials and the mechanisms of their impact on individual cells as well as on entire organisms. The particular need to raise public awareness of the main sources of exposure to nanoparticles should also be highlighted. This paper presents the main sources and possible routes of exposure to metal nanoparticles and metal oxides. Key elements of research on the impact of nanoparticles on organisms, that is, in vitro tests, in vivo tests and methods of detection of nanoparticles in organisms, are presented.

## 1. Introduction

The rapid development of nanotechnology causes exposure to organisms of particles of 1–100 nm [1]. Due to their physicochemical properties, nanometric substances are used, among others, in the cosmetics, electronics and textile industries, biotechnology, environmental engineering and everyday products, as well as in medicine as imaging diagnostics substances and as drug delivery systems [2,3,4].

**Figure 1:**
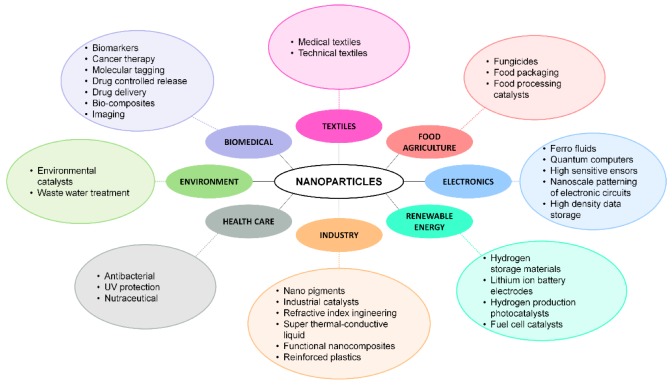
Spectrum of nanomaterial applications.

Nanopharmacology is the fastest growing field of nanomedicine [5]. Over the past two decades, nanopharmaceuticals have been used to treat cancer, diabetes, asthmatic diseases, allergies and various types of infection [6,7,8,9,10]. Nanoparticles are also an effective tool for delivering biopharmaceuticals. Pathological cell receptors bind to ligands surrounding nanoparticles [11,12] The surface and size modification of ultra-small particles allows them to be used as drug delivery systems in therapies targeted at specific tissues, cells and cell receptors [13,14,15]. The development of new treatment methods based on nanoparticles results from the reduction of the effectiveness of antibiotic therapy due to the emergence of antibiotic resistant strains. In such cases, copper, gold and silver nanoparticles with antibacterial properties are successfully used [16,17,18,19]. Apart from pharmaceuticals on the market, cosmetic preparations containing nanoparticles are available. Sunscreen preparations containing nanoparticles of titanium or zinc have been popular since the 1980s [20]. Currently, metal or metal oxide nanoparticles can be found in a wide range of products that are applied directly to the skin, such as disinfectants, creams, shampoos, antiperspirants and toothpastes [21,22,23]. Figure 1 presents a broad spectrum of nanomaterial applications.

The seemingly favorable properties of nanomaterials, as a result of their small size, can have a detrimental effect on soil and water, and thus on living organisms. In the case of uncontrolled exposure to contact with nanoparticles, they can enter the body through the digestive system, respiratory system and skin, and then accumulate in various organs [24,25]. They can also easily enter the blood through the capillaries and lymphatic endothelium, and thus enter the brain [26,27]. 

Nanomaterials’ unique features, the broadening spectrum of applications and the general availability of nanotechnology products obscures safety issues for manufacturers, consumers and the environment. Exposure of living organisms to contact with nanometric particles does not always have a positive impact on them. The widespread use of nanomaterials causes leakage of ultra-fine particles into the environment in an uncontrolled manner, and their presence is noticeable in water and dust suspended in the air [28]. Due to their small size, nanoparticles easily enter the blood, and therefore accumulate in organs [29]. 

## 2. Forms of Exposure to Nanoparticles

### 2.1. Drug Carriers

Nanoparticles are successfully used as drug delivery systems, improving the effectiveness of conventional treatments. Drug carriers are used to deliver drugs in a controlled manner in many targeted therapies by combining metallic, organic, inorganic nanostructures, polymers, etc. [14,30]. The drug carrier must have bioavailability, which is influenced by several factors including the method of administration, absorption rate, and ease of metabolizing the pharmaceutical [31,32]. 

The supply of drugs on a nanometric carrier has gained popularity in the last decade. This method makes it possible to reach tissues whose treatment is difficult with traditional methods. In addition, the nanocapsulated formulation has fewer adverse effects and is delivered in a controlled manner. The effectiveness of therapy is influenced by the size of nanoparticles, encapsulation efficiency, degree of drug release and surface charge of the drug on a nanometric support [33]. Currently, carriers are used to treat chronic diseases such as pulmonary tuberculosis, diabetes, cancer, atherosclerosis, Parkinson’s disease, asthma and Alzheimer’s disease [34]. 

Drugs can be delivered to the body orally, ophthalmically, nasally, through the skin, parenterally or through the respiratory tract. The easiest method of application of the drug from the patient’s point of view is orally. The low pH of the stomach and the presence of mucus prevent the development of innovative orally administered pharmacological preparations. To improve the bioavailability of the drug, it is enclosed in polymeric nanoparticles [30]. Due to the small particle size, their increased surface area and high activity, the bioavailability of the preparation increases during oral administration [35]. 

Pharmacological preparations can also be administered through the nasal mucosa, which is characterized by a large surface area and numerous blood vessels by which the administered substance is transported to the circulatory system [36]. Mucolytics, antibiotics and mucosal decongestants are administered through the nasal cavity. The use of nanotechnology has made it possible to administer anti-cancer drugs, preparations affecting the central nervous system, vaccines, and proteins through the nose. By nasal administration of a nanoparticle filled with insulin, its half-life and stability are increased, and thus, the efficiency of action increases [34]. In turn, the application of the vaccine via the nasal mucosa allows delivery of antigens directly to lymphatic tissues. The use of nanotechnology overcomes the blood–brain barrier and allows delivery of the drug to the brain. During conventional therapies, endothelium limits the permeability of pharmacological agents, whereby the therapeutic concentration of the pharmacological substance in the brain is insufficient [37]. The ability to easily administer the drug through the nose and transport it to the brain promises to successfully treat migraines, epilepsy, brain tumors and schizophrenia [38]. 

Pharmacological preparations are also administered directly to the affected tissue [39]. This method is most notably used in the fight against cancer, where nanoparticles are an effective tool in localized chemotherapy. By delivering the therapeutic preparation locally, it is retained longer in the affected organ, which reduces the undesirable effect on the rest of the body [40,41,42]. Hu et al. confirmed the effectiveness of arsenic trioxide nanoparticles in the fight against malignant liver cancer. The studies were carried out on mice treated with arsenic trioxide nanoparticles directly into the tumor. Inhibition in disease development and greater lactate dehydrogenase release were observed [43]. Iron oxide nanoparticles are also used in cancer therapies. Nanoparticles enter cancer cells, leading to their death or apoptosis [44], and also regulate the transcription of pro-inflammatory genes, resulting in severe cell genotoxicity and subsequent death. This effect is used in the treatment of cancer; however, the molecular mechanisms responsible for nanotoxicity are not yet known, which is a barrier to the use of nanoparticles in vivo [45]. 

Medicinal substances can also be applied to the skin. The basic role of the skin is to protect the body against external factors. However, the nanometric particle size means that the skin barrier is not an obstacle for them. These particles are transported through the stratum corneum, hair follicles and sebaceous glands [46,47]. For people with a skin barrier dysfunction (such as psoriasis, atopic dermatitis, acne), whose skin continuity may be interrupted for various reasons, the penetration of nanoparticles into the deeper layers of the skin is even easier [48,49,50]. Nanoparticles move between corneocytes arranged in a “brick-like” manner in the lipid layer [51]. The efficiency of penetration of nanoparticles through the skin depends on their size [52]. For particles larger than 45 nm, penetration through the skin is difficult, particles between 21 and 45 nm penetrate only through damage to the skin and smaller particles of the order of 4–20 nm penetrate both intact and damaged skin. Nanoparticles smaller than 4 nm can even penetrate through intact skin to deeper layers [53]. In addition, the overall composition, shape and type of preparation in which these particles are situated affect the ability of nanoparticles to penetrate the skin [52,54]. Musazzi et al. [55] studied the effect of the consistency of preparations with iron oxide nanoparticles on their penetration into the deeper layers of the skin. The degree of penetration of nanoparticles depended on the type of preparation applied to the skin (cream or gel). The highest penetration of iron oxide nanoparticles was observed for cream. This is undoubtedly affected by the physical stability and the shape of nanoparticles in various environments [56,57]. Tak et al. presented assumptions regarding the impact of the shape of silver nanoparticles on skin penetration, diffusion coefficient as well as speed and depth of penetration. Silver nanoparticles of longitudinal shape penetrated deeper into the stratum corneum [51].

### 2.2. Diagnostic Imaging

Nanotechnology is also successfully used in diagnostic imaging to increase the effectiveness of magnetic resonance imaging, computed tomography, ultrasound techniques and fluorescence imaging techniques [58,59,60]. Conventional contrast media contain gadolinium iodine complexes or barium sulfate which have the disadvantage of having a relatively short half-life and poor efficacy in overweight patients [61,62]. Lipid-based nanocarriers can be used in Raman spectroscopy; in this case, contrast is based on molecular vibrations in the sample [60]. Silver and gold are the most popular nanoparticles used in surface enhanced Raman spectroscopy. The targeted actions of biomarkers are obtained through the interaction of various nanostructures with other nanoparticles, biomolecules or coating materials [62]. As the Raman imaging technique develops and its sensitivity increases, it is anticipated that imaging of nanocarrier placement in living systems in real time will be possible in the future. Nanoparticles are used in contrast agents due to their biocompatibility, high X-ray absorption coefficient and the ability to target a specific pathological tissue [63,64]. In addition, the nanoparticle contrast agent administered during the study improves the quality and resolution of the image, which allows earlier detection of lesions in the body. The size of the contrast agent’s nanoparticles has an impact on how quickly it is removed from the body; larger particles are removed faster. Nanoparticles larger than 200 nm are sequestered by the mononuclear phagocyte system found in the spleen, while particles smaller than 10 nm are usually invisible to the organs of the phagocytic system [65,66]. 

The rapid development of nanotechnology has made it possible to obtain contrast agents based on nanoparticles of various types and structures [67]. Gold is a suitable metal for use in contrast media. After achieving colloidal stability gold nanoparticles have the ability for active targeting [50,68,69]. Gold nanoparticles and gold nanoshells, so-called plasmon-resonant nanoparticles, are successfully used in optical coherence tomography, a method that facilitates the evaluation of the choroidal and retinal microvasculature and in optical coherence tomography [70,71,72]. Contrast containing nanometric bismuth has similar properties to contrast containing gold and is much cheaper [73,74]. Iron oxide nanoparticles are also used as a contrast agent with strong supermagnetic properties. It has no toxic properties and it is metabolized to hemoglobin; however, it is believed that the high content of iron is toxic [75,76,77].

### 2.3. Uncontrolled Exposure to Nanoparticles

Widespread use of nanoparticles in commercial products results in them spreading into the environment. The presence of nanoparticles is observed in air, soil and water. As nanotechnology is a relatively young field of science, there is currently insufficient research into the effects of long-term human exposure to nanoparticles. The general effect of metals on the health of living organisms is widely known; however, changing the size of materials to the nano scale results in a modification of their properties. Nanoparticles found in everyday items can be released into the environment [78]. Sports clothing containing silver nanoparticles with bactericidal properties is gaining popularity, but textiles containing nanoparticles release some during washing. Benn and Westerhoff [79] conducted a study on the release of silver nanoparticles from clothing into water. For this purpose, they used socks made of various materials which were then left in contact with water for a period of one or 24 h. The rate of release of nanoparticles from the socks varied and depended on the production methods and the type of material from which they were made. The degree of release of nanoparticles from a material depends on the frequency of washing, the type of surfactant, the pH of the water and the number of oxidizing agents [79,80]. It is estimated that during the entire lifetime of a textile material, it loses up to 10% of its weight, which is influenced by both the washing process and friction during use, suggesting that the release of solid particles may play a dominant role. The research also shows that in one wash cycle, up to 75% of nanoparticles can be released in the washing machine, which then goes into the sewage system with the water [81]. 

The presence of nanoparticles in the natural environment is a threat to microorganisms found in soil, such as bacteria and fungi, which in turn can lead to irregularities in the functioning of the ecosystem [82]. Nanoparticles are retained in soil more than in water and air. Their source is sewage sludge used as fertilizer, landfills, various industrial leaks and metals introduced into the soil in the bioremediation process [83]. 

Nanoparticles in the air easily enter the respiratory system [84]. These particles come largely from industrial processes, and can pose a threat to people in contact with them [85]. The mechanism of selective metal transport to the olfactory bulb through the olfactory nerve was determined based on research on mammals. According to this mechanism, the metals are taken from the primary olfactory neurons, from which they go to the olfactory bulb and even on to other areas of the brain [86]. Scientific research confirms that the uncontrolled absorption of metal nanoparticles through the respiratory tract can also result in cardiovascular diseases [87].

## 3. Exposure of Metal and Metal Oxides Nanoparticles

Silver nanoparticles (AgNPs) are one of the most popular examples of inorganic nanoparticles. AgNPs show significant biocidal activity, partly due to the prolonged release of silver ions. The broad spectrum of applications of this metal causes uncontrolled migration of AgNPs into the ecosystem. It is estimated that 7% of AgNPs reaching wastewater treatment plants leave the facility in sludge [88]. However, their further fate is largely unknown and depends on the sediment management system of the region, including storage, incineration and land use. Gold nanoparticles (AuNPs) are the most widely used inorganic nanomaterials in medicine. They are used as biolabels, biosensors and as drug carriers. Titanium oxide nanoparticles are used as biomedical ceramic implants and, together with zinc oxide, they work well as drug carriers [89]. 

The wide toxicity effect of nanoparticles, especially metal and metal oxides, can be due to the different characteristics of these particles. Both positive and negative properties of nanoparticles are influenced by the size of the particles used, their size distribution, surface area, composition, stability, the presence of additives, and many other factors [90]. Therefore, to characterize the properties of nanomaterials in terms of their possible negative impact on the environment, a variety of measurement methods should be used [91].

When analyzing materials of nanometric size the undoubted problem is the size of the particles, which makes it possible for them to penetrate the cells and agglomerate in organs in different parts of the body. In addition, the low concentration of nanoparticles found in cells, tissues, organs, and organisms presents significant difficulties in detecting them. Although there are many general methods dealing with quantitative and qualitative characteristics of nanoparticles, these methods require prior preparation of the sample, including the use of dissolution, digestion, mineralization, etc. [92]. In the case of in vitro methods, degradation of tissue and cell samples is possible, which makes the characterization of nanoparticles contained therein easier. In the case of living organisms, it is not possible to remove tissues exposed to the presence of nanoparticles, especially if it is not known in which parts of the body accumulation occurs. Therefore, it is first necessary to develop and use non-destructive methods that would facilitate determination of the presence of nanoparticles directly in cells. Secondly, the methods should enable testing of the concentrations throughout the body and on this basis assess the degree of exposure to nanoparticles or specify the locations of accumulation in organisms. Another difficulty in researching nanoparticles in the body is the fact that they undergo biotransformation processes, which cause a change in their possible toxic effect by redox processes, gradual dissolution, sulfidation, aggregation and adsorption of macromolecules and ions, as a result of which they can be transported and accumulated throughout the body [88].

## 4. Characteristics of the Field of Metal and Metal Oxide Nanoparticle Research

To assess the probability of harmful effects of nanoparticles on human health and the environment, a proper risk assessment is necessary. For risk assessment, the identification of the effects caused by the particles is required, as well as dose–response relationships [93]. For nanoparticles, information related to the possible effects on organisms is very limited. In connection with the above, it is necessary to conduct a number of indirect studies, from which it will be possible to develop models of the impact of nanoparticles on cells, tissues and organs as much as possible, and in the final stage to affect the entire organisms of both animals and humans [94]. To this end, research methods on nanoparticles and the effects of their impact on the environment can be divided into four groups: Methods for determining the physicochemical properties of nanoparticles, in vitro methods, in vivo methods, and ex vivo methods.

### 4.1. Methods for Testing Physicochemical Properties

The first step in understanding the structure and characteristics of products containing metal and metal oxide nanoparticles is the analysis of the physicochemical properties of nanoparticles, including UV-VIS spectroscopy, dynamic light scattering (DLS), X-ray photoelectron spectroscopy (XPS), photoluminescence spectroscopy [95,96], scanning electron microscopy (SEM), transmission electron microscopy (TEM), atomic force microscopy (AFM), high-resolution transmission electron microscopy (HRTEM) [97], X-ray powder diffraction (XRD), inductively coupled plasma mass spectrometry (ICP-MS) [98]. Non-cellular assessment of nanoparticle stability, including interaction with proteins and pro-oxidative activity, is also significant [99]. 

Imaging methods are used for qualitative analysis of samples containing nanoparticles. The main imaging methods of nanoparticles extracted from the body include TEM and SEM methods are usually combined with the X-ray dispersion spectroscopy (EDX) method, which makes it possible to determine the elemental and phase composition of the analyzed materials [100]. Using nanoparticle imaging, it is possible to assess the actual size of nanoparticles and the degree of their agglomeration, shape and structure. It is important that all the methodologies described allow for simultaneous visualization of nanoparticles and the assessment of changes in cells and tissues that are essential in toxicopathological studies [101].

Microscopic imaging has considerable limitations, one being the fact that they are destructive methods. In addition, it is necessary to properly prepare the sample as it is not possible to study nanoparticles directly in the body [102]. In the case of animal tests, necropsy is performed to isolate the organs in which the probability of agglomeration of nanoparticles is highest. Therefore, the use of the methods presented works only to predict, based on animal studies, which parts of the human body are most exposed to the action and accumulation of nanoparticles [103]. 

AFM and scanning tunneling microscopy (STM) analysis can be used for biological materials. In this way, there is no damage to the biological matrix (cells and tissues), which is the case with electron microscopy. Both devices belong to the group of microscopes with a scanning probe, which allows the imaging of scanned samples. Surface topography is the configuration of the surface, taking into account its shape and the presence and mutual location of its characteristic points, including the presence of nanoparticles. The main application of the AFM and STM methods is an attempt to explain the interaction of nanoparticles on individual cells. Consequently, it is possible to characterize how cells containing nanoparticles change their structure and vital functions [104]. The accumulation of nanoparticles can be predicted in further studies by determining changes in tissues.

Imaging methods allow the qualitative properties of nanoparticles to be assessed, particularly ICP-OES, ICP-SIMS, ICP-MS, etc., and quantitative methods are also used. Gray et al. confirmed the possibility of using the ICP-MS method for single particles (spICP-MS) for the quantitative analysis of gold and silver nanoparticles of 60 and 100 nm [105]. In addition to the number and mass concentration for samples containing metal nanoparticles in less than 10 μg/L, this technique enables the determination of particle size distribution.

A different technique that also makes it possible to characterize nanoparticles quantitatively is nanoscale secondary ion mass spectrometry (nanoSIMS). This method uses a beam of energy ions to bombard the sample. Particles from several upper atomic layers of the surface are removed, which releases the so-called secondary ions. These ions are then sorted based on energy and dispersed in a mass spectrometer to produce maps providing information on elemental or molecular distribution in the sample. The nanoSIMS technique is one of the most powerful tools for quantifying the distribution of elements in the body at the cell level. NanoSIMS is reported to perform nanoparticle analysis in biological samples. The limitation of the technique is the difficulty in analyzing some elements, especially those with poor secondary ion performance (Zn, Cd and Mn). It is also a destructive technique, which can be a disadvantage for various operations. By applying high pressure and freezing followed by thawing, cellular structures remain preserved [106].

### 4.2. In Vitro Analysis

In vitro methods allow the description of the impact of nanoparticles on the cells of individual strains of microorganisms, making it possible to learn how nanoparticles are transported into cells, the mechanisms of interaction of nanoparticles on metabolic pathways in cells and the mechanisms causing their damage or death. Soni et al. determined the toxicity of commercial zinc oxide nanoparticles (ZnO NPs) onto *Pseudomonas* sp. bacteria, human promyelocytic leukemia cells and peripheral blood mononuclear cells. Toxicity was assessed by measuring cell growth, viability and protein expression in a bacterial cell. Studies have shown the toxicity of nanoparticles to human cells. Bacterial growth and viability decreased with increasing ZnO NP concentration. The main ribosomal proteins, together with the alkyl hydroperoxide reductase, were up-regulated after exposure to ZnO NPs. The results indicated oxidative stress as the main cause of bacterial toxicity [107].

Although nanoparticles have brought significant benefits in various fields of science, their harmful effects have been noted, suggesting the need for appropriate risk assessment. In vitro research on human cells makes it possible to determine the mechanisms of reaction between nanoparticles and selected cells [108]. Williams et al. studied the effect of exposure to silver nanoparticles (AgNPs) of various sizes (10, 20, 75 and 110 nm) and doses (20 and 100 µg/mL) on the permeability of human T84 colon epithelial cells, which serve as an in vitro model of human intestinal epithelium. The results showed that the effect of AgNPs on T84 epithelial cells was dependent on size and dose, with 10 nm of AgNPs causing the most significant changes. Exposure to AgNPs significantly affected the expression of genes involved in proliferation, cell signaling, endocytosis and cell–cell adhesion. Such effects may potentially threaten the integrity of the intestinal epithelium and, as a consequence, disrupt the barrier functions of the gastrointestinal tract [109]. Ng et al. studied ZnO NPs toxicological profiles in MRC5 human lung fibroblasts in vitro. Based on the results, it was found that there was a significant release of extracellular lactate dehydrogenase and a decrease in cell viability in MRC5 lung fibroblasts treated with ZnO NPs, which indicated cell damage and confirmed the increased cytotoxicity and genotoxicity associated with oxidative stress in human lung fibroblasts [110].

### 4.3. In Vivo Analysis

In vivo methods determine health effects, mainly in animals, after exposure to nanoparticles depending on the route of exposure. The studies pay attention to the location of nanoparticle accumulation in individual organs, the impact of nanoparticle concentration causing negative effects, including toxicity tests on organs, for example, lungs, liver, spleen, brain, kidneys, nervous system and tissues and secretions, skin, mucous membranes and blood [111]. By applying this approach, it is possible to assess the risk of nanoparticles for entire organisms by examining the interrelationships of individual systems and vital functions. In vivo research is currently one of the best sources of risk assessment for nanomaterials.

Based on in vivo studies, it is possible to describe the pathways of nanoparticles through cells as well as their interactions with cells. Liu et al. described possible excretion routes of nanoparticles, for example, silver, gold and magnetic oxide nanoparticles on mouse organisms. The authors injected nanoparticle suspensions into the circulatory system and examined the content of nanoparticles both in urine and feces, as well as after death in gastric and intestinal cells. Based on this research, they observed that nanoparticles are mainly excreted from mouse organisms through the digestive system, and nanoparticles accumulated inside the gastric parietal cells and in the goblet cells of the whole intestinal tissue. Despite particle deposition in selected cell types, no organ damage was observed. Knowledge about which cells accumulate nanoparticles can be used when administering drugs, with the ability to precisely control the concentration of nanoparticles [112]. Despite the widespread use of magnetic nanoparticles in diagnostics and as drug carriers, there is no information on their life cycle in organisms. Van de Walle et al. investigated their transformations upon internalization in cells with different functions. Stem cell research shows the possibility of the degradation of magnetic nanoparticles, transforming them into new iron compounds. Research has provided a better understanding of how cells can interact with nanoparticles [113]. Comparing the results of in vitro and in vivo analysis, George et al. studied the kinetics of silver nanoparticles absorption by human skin cells. Based on the research, it was found that silver nanoparticles penetrate the stratum corneum and enter the reticular dermis. The agglomeration of nanoparticles was also observed, which prevented their penetration into the circulatory system [114]. 

### 4.4. Ex Vivo Analysis

Despite the usefulness of information obtained from in vitro and in vivo analyses, it is not possible to obtain a complete picture of the effects of nanoparticles on organisms without ex vivo tests. To determine the relationship between the dose and route of nanoparticles entering the body and its response, research conducted in the real environment of the human body is important [93]. For such assessments it is crucial to introduce new techniques for nanoparticle characterization. They enable the quantitative and qualitative measurement of nanoparticles in the body. To this end, tests are initially carried out on the analysis of body fluids and tissue fragments on the basis of which the organism’s exposure to nanoparticles is assessed.

#### 4.4.1. Penetration of Nanoparticles Through the Circulatory System

After entering the body, regardless of the routes to exposure, nanoparticles are transported through the central circulatory system to various organs and tissues, including the liver, spleen, heart, kidneys and brain [85]. Animal studies have shown a low but detectable level of inhaled radio-labelled particles into these organs. The presence of nanoparticles in organs suggests their distribution by the circulatory system [115]. Rothen-Rutishauser et al. studied the effects of gold nanoparticles and titanium oxide entering human red blood cells. Based on the results, it was found that the particles can penetrate the red blood cell membrane by a mechanism previously unknown, other than by phagocytosis and endocytosis [116].

The analysis of blood samples allows a determination not only of the presence of nanoparticles in the body, but also the analysis of changes in the body fluids transporting accumulated nanoparticles themselves. Chupani et al. investigated the relationship of ZnO NPs supplied with food by studying common carp *(Cyprinus carpio)*. The authors analyzed their blood, using methods such as erythrocyte count, leukocyte count, hematocrit, hemoglobin, mean blood hemoglobin, mean blood volume, glucose, triglycerol and cholesterol, total proteins, aspartate aminotransferase and alanine aminotransferase [117]. Despite exceeding the limit values for some parameters, no effects on hematology, blood biochemistry or lipid peroxidation levels were found during the exposure period. There was no significant increase in the zinc content at the end of the experiment in any of the tested organs, which confirmed the need for the widest possible analysis of the impact of nanoparticles to obtain a full picture of the body’s dose-response system.

#### 4.4.2. Penetration of Nanoparticles Through the Skin

Skin is the first and main barrier against the entry of nanoparticles into the body. The increase in the share of preparations containing nanoparticles in their composition, mainly titanium oxide and zinc oxide, is constantly increasing [104]. The nanoparticles contained in the preparations can penetrate the skin into their deeper layers as well as penetrating the circulatory system. It is necessary to conduct analyses aimed at determining the level of nanoparticles entering the body, with a short exposure time in a high concentration, which may have more harmful long-term exposure effects even at low concentrations of nanoparticles [118].

The effect of zinc oxide nanoparticle penetration through the skin barrier was studied by Leite-Silva et al. They did not observe significant penetration into live epidermis and no cellular toxicity was detected. This may have been due to the relatively large particle size (75 nm) tested. The studies conducted on 20 volunteers were compared with the results conducted on human skin cells. In simultaneous in vivo studies, they were imaged at two key layers: Stratum granulosum (SG) and stratum spinosum (SS). The authors developed a quantitative method for determining ZnO in the skin using a slow, luminescent, component duration of ZnO emission. A slight increase in the penetration of the uncoated ZnO epidermis was observed, which was attributed to the accumulation of nanoparticles in the sebaceous glands [119]. In another study, Leite-Silva et al. investigated the impact of the daily use of sunscreen products based on ZnO NPs on the penetration of nanoparticles deep into intact and damaged skin. Multi-photon tomography with fluorescence imaging was used to evaluate non-invasive ZnO penetration and metabolic changes in cells that may indicate toxicity. It was found that the ZnO NPs did not penetrate the living epidermis of intact or damaged skin of volunteers; however, the risk of nanoparticle penetration with prolonged use of similar preparations could not be excluded. Real-time non-invasive analysis of nanoparticles was also confirmed. Cellular toxicity can be measured by MPT-FLIM multiphoton tomography fluorescence lifetime microscopy using the phenomenon of apoptosis, which is associated with an increase in the average time of the NAD (P) H fluorescence effect [118].

Due to the possibility of penetration of nanoparticles of about 10 nm through the skin, Schulz et al. examined the skin absorption of three titanium oxide (TiO_2_) preparations with variable characteristics: Particle size (10–15 nm, 20 nm and 100 nm), shape (cubic or needle) and hydrophobic/hydrophilic. The formulations were applied topically in an oil-in-water emulsion to the skin of the forearm of volunteers for six hours. The effect of the presence of nanoparticles was studied by skin biopsies, and then the samples were examined using a scanning electron microscope to visualize the distribution of particles in the layers of the skin. Based on the results, no nanoparticles were found outside the outer horny layer of the epidermis of 10–15 nm nanoparticles [120]. Sadrieh et al. also studied the effect of TiO_2_ nanoparticle size on skin penetration. The tests were carried out on miniature pigs and an extended application time of the cream with nanoparticles was assumed (four weeks). After this time, the pigs were killed and skin samples, lymph nodes, liver, spleen and kidneys were taken from them. In the samples, titanium levels were determined by ICP-MS. The epidermis from miniature pigs treated with TiO_2_-containing sunscreens showed an increased content of titanium, while the content of titanium in lymph nodes and livers did not increase above the value recorded in the control animals [121]. The results of the tests indicated that there was no significant penetration of ZnO and TiO_2_ nanoparticles through the intact epidermis in both animals and humans; however, it was necessary to conduct tests with prolonged exposure to nanoparticles.

A number of experiments have been described in the literature on skin penetration of nanoparticles [122,123,124,125]. These studies were carried out on metal nanoparticles or metal oxides, and the particles were characterized by various sizes and shapes and were dispersed in various centers, which undoubtedly influenced the results of the experiment.

Different conclusions regarding the penetration of nanoparticles through the skin were described by Larese et al. [126]. In vitro studies looked at the penetration of AgNPs through damaged and intact skin. The degree of human skin penetration and verification of the location of AgNPs was conducted using a transmission electron microscope (TEM). Based on experimental data, it was shown that the absorption of AgNPs by intact and damaged skin was very low but detectable, which proves that silver in the form of nanoparticles is able to penetrate damaged skin. 

#### 4.4.3. Penetration of Nanoparticles Through the Digestive System

The results of morphology analysis are a convenient way to detect changes in the body. Nanoparticles of metals and metal oxides determined in urine and feces are the result of absorption mainly by the digestive tract. It was found that oral silver is absorbed in the range of 0.4%–18% in mammals and a significant number of nanoparticles delivered to the body are excreted [127]. Furchner et al. found that the fecal excretion rate of nanoparticles after oral administration exceeds 90% for all animals tested [128].

Jones et al. conducted in vitro tests on human cells to determine the extent of absorption of TiO_2_ nanoparticles in the gastrointestinal tract. They also determined the extent of absorption of nanoparticles on living organisms in ex vivo studies involving nine volunteers. Volunteers received a single oral dose of 5 mg/kg TiO_2_ dispersed in water. The tests were carried out for three particle sizes: 15, 100 and 5000 nm, at intervals of at least four weeks between each dose of nanoparticle suspension. Analyses were made on the basis of titanium content in urine and blood samples, complete blood counts and liver function tests. Studies have shown that a small amount of TiO_2_ is absorbed from the gastrointestinal tract after oral administration. Based on studies comparing the uptake of nanoparticles and particles of micrometer size, no evidence was found that nanoparticle TiO_2_ is more susceptible to absorption in the intestine than particles of micrometer size, and none of the volunteers exceeded the normal clinical range [129].

Based on in vivo tests, it is possible to predict the transport route and accumulation site of nanoparticles in human organisms. Cho et al. evaluated the distribution and excretion of TiO_2_ and ZnO nanoparticles after oral administration to rats. Blood, tissue (liver, kidney, spleen and brain), urine and feces samples were collected during autopsy. Based on the research, it was found that TiO_2_ nanoparticles had extremely low absorption compared to the absorption of ZnO NPs. A significantly higher accumulation of ZnO NPs was observed compared to TiO_2_, especially in the liver and kidneys. In addition, much higher levels of ZnO in the urine were observed, in contrast to TiO_2_. Higher ZnO absorption than TiO_2_ nanoparticles may be caused by the rate of dissolution of these substances in acidic gastric fluid [130].

Due to the high production of TiO_2_ and the increasing use of TiO_2_ nanoparticles for consumer and food products. Hering et al. examined the concentration of nanoparticles in post-mortem human livers and spleens. By using low-level inductively coupled plasma-high resolution mass spectrometer (ICP-HRMS), it was possible to quantify both total titanium (Ti) and TiO_2_ particles. The presence of TiO_2_ in the tissues, as well as their qualitative assessment, was carried out using scanning electron microscopy and X-ray energy dispersion spectrometry. The presence of TiO_2_ in human liver and spleen was confirmed, stating that a minimum of 24% of the particles had a size less than 100 nm. The levels were below the doses considered safe for animals. It is believed that the presence of Ti and TiO_2_ may have been due to liver damage due to oral exposure to titanium nanoxide. Based on the tests performed, a risk to health caused by oral exposure to TiO_2_ cannot be excluded [131].

#### 4.4.4. Penetration of Nanoparticles through the Respiratory System 

The respiratory system is the easiest pathway that nanoparticles suspended in the air can enter the body. Beckett et al. compared the dependence of exposure to ZnO NPs on the respiratory, hematological and cardiovascular systems. These studies have a special contribution to the subject because they were conducted with healthy organisms exposed to nanoparticles in various forms. A group of healthy people inhaled 500 μg/m^3^ of suspended ZnO NPs for two hours and after the study no disturbing symptoms, such as a change in body temperature or white blood cell count, were noticeable after exposure to 5.0 and 2.5 mg/m^3^ of ZnO NPs [132].

In the event that a potential source of exposure is identified, including in industrial sectors, it is possible to determine the concentration of nanoparticles in suspended dust in the air. A number of methods are used for this, including cascade impactor, tapered element oscillating microbalance (TEOM) used in air quality monitoring stations, electrical low pressure impactor (ELPI) or nanoparticle surface aerosol monitor (NSAM) [133]. Most devices, however, are used to assess the presence of nanoparticles in a particular room and are not intended for individual use. An example of a device for individual measurements is the cascade impactor enabling the sampling and analysis by individuals who are particularly exposed to contact with nanoparticles. It provides an aerosol mass distribution in the gas relative to the aerodynamic diameter of the collected particles. An NSAM is a device that measures the surface concentration of an aerosol that settles in the tracheobronchial or alveolar airway. This device is mainly used for static purposes, but it is possible to use portable devices for individual use.

Fissan et al. investigated the operation of the electric aerosol detector, demonstrating the ability to assess the behavior of nanoparticles under similar lung deposition conditions in various areas of the human respiratory system. The apparatus was tested with monodisperse AgNPs of 10 to 100 nm at an ion trap voltage of 20 to 200 V. Despite the simplifications used in adopting a spherical model of nanoparticles and the use of idealized lung deposition curves, the results obtained from the detector allow only a preliminary assessment of human exposure absorption of nanoparticles through the respiratory system. The advantage of the method presented is the possibility of obtaining additional information, such as the dose of nanoparticles per unit of lung surface or lung mass [134].

Inhalation of metal oxides in nanometric form can cause systemic diseases, alveolar epithelial damage and inflammatory response to endothelial cells. Bengalli et al. used the in vitro air–blood barrier (ABB) model as a tool to explain the biological mechanisms underlying the potential effects of nanoparticle inhalation. The model used consisted of the co-culture of Transwell pulmonary epithelial cell line (NCI-H441) and microvascular lung endothelial cell line (HPMEC-ST1.6R). These models were created to analyze the importance of the interaction of different cell types to different responses after exposure to ZnO. The results showed that the in vitro-ABB model used indicates the interaction between lung epithelium and endothelium in inducing a biological response and the role of endothelial dysfunction after inhalation of nanoparticles [135].

## 5. Risk Analysis for Exposure to Nanoparticles

The use of nanoparticles in packaging and food products as carriers of drugs or excipients and in a number of consumer products means that the degree of exposure of nanoparticles to plants, animals and people in the near future will increase. Analysis of the paths through which nanoparticles get into the organisms is very important when assessing the possible site of their accumulation as well as the concentration in which they may occur [116]. Possible routes of exposure to metallic and metal oxide nanoparticles are inhalation, penetration through the skin and penetration of particles through the digestive system. Exposure to the absorption of nanoparticles through the respiratory system is negligible and applies in particular to people present in a place of nanomaterial production [134]. The penetration of nanoparticles through the skin is one of the main pathways for particles to enter the body. The reason for this may be products containing nanoparticles in their composition that are in constant contact with the skin, including cosmetics and dermatological preparations, textiles, dressing materials. In this case, nanoparticles can enter the body and accumulate in the liver or even the brain [118]. However, research confirms that the penetration of nanoparticles through the skin is low. In addition, by limiting contact with preparations containing metal nanoparticles and metal oxides, there is no exposure to their harmful effects. The sources of nanoparticles found in the digestive system are medicines and medicinal substances, but also food products stored in packaging modified with nanoparticles. Due to the antimicrobial activity of AgNPs, they also began to be used as an additive to materials in contact with food, including kitchen utensils and storage containers [136]. Manufacturing products modified with nanoparticles intended for food processing and storage without tests verifying their non-toxicity, the degree of their accumulation in cells and the percentage of penetration of nanoparticles into food, is risky [85].

## 6. Conclusions

Metal and metal oxide nanoparticles offer many benefits, but their presence in the environment is not indifferent to organisms. Increasing the production scale of nanomaterials contributes to the uncontrolled introduction of nanometric substances into the environment, which raises concerns for health and safety. The methods of penetration of nanoparticles into living organisms, assessment of the toxicity of nanomaterials, as well as determination of their negative impact on the environment are growing areas of interest. The development of nanotechnology, research methods enabling the recognition of nanoparticles, and the mechanisms of their action increases awareness of the risks arising from the exposure of organisms to nanomaterials.

Due to the presence of nanomaterials in an ever-wider range of products, it is necessary to be aware of possible exposure to them, as well as to conduct research aimed at approximating the mechanisms of nanoparticle interactions with living organisms.
